# Supplementary material for: Aberrant E-I Balance and Brain Criticality in Major Depressive Disorder
Source: bioRxiv. 2025 Oct 20:2025.10.19.683280. Preprint. [Version 1] doi: 10.1101/2025.10.19.683280 (PMC12633248; doi:10.1101/2025.10.19.683280)
Supplement: 1 [file NIHPP2025.10.19.683280V1-supplement-1.pdf]

bioRxiv preprint doi: https://doi.org/10.1101/2025.10.19.683280; this version posted October 20, 2025. The copyright holder for this preprint (which was not certified by peer review) is the author/funder, who has granted bioRxiv a license to display the preprint in perpetuity. It is made available under aCC-BY 4.0 International license.

Supplemental Information

EEG acquisition (MDD)

Clinical EEG was collected at the Butler Hospital TMS Clinic using a 64-channel ANT Neuro system (EEGO software, 10–10 montage; CPz reference, AFz ground). Signals were recorded with eyes closed, and no online filters were applied. For the present study, we analyzed the pre-stimulation baseline obtained prior to the first TMS session of each patient's earliest treatment series. The raw data were acquired at 2000–2048 Hz for 5–10

bioRxiv preprint doi: https://doi.org/10.1101/2025.10.19.683280; this version posted October 20, 2025. The copyright holder for this preprint (which was not certified by peer review) is the author/funder, who has granted bioRxiv a license to display the preprint in perpetuity. It is made available under aCC-BY 4.0 International license.

minutes and were subsequently resampled to 512 Hz and trimmed to ~5 minutes of artifact-free resting EEG per subject by the data provider. For analysis, each ~5-minute recording was then segmented into five contiguous 1-minute epochs and re-ordered in a fixed sequence (1–3–5–2–4) to construct the final time series, aligning the epoch structure with the 1-minute block design used for HC data. To maximize the number of patients included in the analysis, recordings shorter than 5 minutes were standardized to a common length of 4.65 minutes by removing 10.6 seconds from both the beginning and the end of each recording.

EEG acquisition (HC)

Resting-state EEG was recorded in the LEMON study using a 62-channel active electrode cap (ActiCAP, Brain Products GmbH, Germany; 10–20 montage) in combination with two additional ECG channels (Babayan et al., 2019). Recordings alternated between eyes-open and eyes-closed conditions in 1-minute blocks for a total of 16 min, beginning and ending with eyes open. During eyes-open periods, participants fixated on a low-contrast cross presented on a gray background.

For the present study, we extracted only the eyes-closed segments to match the acquisition protocol used in the MDD patient sample. The 1-minute eyes-closed epochs were concatenated, down-sampled from the original 2,500 Hz to 512 Hz (to align with the patient recordings) and then trimmed to ~5 minutes per subject. To match the data length used for the HC and MDD recordings, an additional 10.6 seconds were removed

bioRxiv preprint doi: https://doi.org/10.1101/2025.10.19.683280; this version posted October 20, 2025. The copyright holder for this preprint (which was not certified by peer review) is the author/funder, who has granted bioRxiv a license to display the preprint in perpetuity. It is made available under aCC-BY 4.0 International license.

from both the beginning and the end of each HC recording, resulting in a length of 4.65 minutes after the preprocessing.

Metrics

Bistability (BiS):

We estimated the probability density function (PDF) of narrow-band EEG power to quantify the bistability of neuronal oscillations. Bistability reflects the tendency of amplitude fluctuations to alternate between high- and low-power states, resulting in a bimodal distribution in critical systems (Freyer, Aquino et al. 2009).

For each channel × frequency band, the analytic amplitude A(t) was obtained using the Hilbert transform after bandpass filtering, squared to yield instantaneous power, and normalized by the median. The empirical PDF of A(t) was then estimated from histograms with 200 equally spaced bins.

To assess bimodality, we compared a unimodal exponential distribution against a bi-exponential mixture, both fitted by maximum likelihood estimation. Model evidence was evaluated using the Bayesian Information Criterion (BIC), and the Bistability Index (BiS) was defined as:

$$\Delta BIC = nBIC_{Exp} - nBIC_{BiExp}$$

$$BiS = \begin{cases} \log_{10}(\Delta BIC), if \Delta BIC > 1, \\ 0, if \Delta BIC \leq 1. \end{cases}$$

For the model fitting, parameters were optimized using the Nelder–Mead algorithm. The two exponents for the bi-exponential model were both initialized to the

bioRxiv preprint doi: https://doi.org/10.1101/2025.10.19.683280; this version posted October 20, 2025. The copyright holder for this preprint (which was not certified by peer review) is the author/funder, who has granted bioRxiv a license to display the preprint in perpetuity. It is made available under aCC-BY 4.0 International license.

value estimated from the single-exponential fit, and the mixing weight was initialized to 0.5. During optimization, we enforced the constraints that the first exponent must be larger than the second and that the mixing weight must lie between 0 and 1.

Following prior work (Avramiea, Diachenko et al. 2025), our computation of nBiC treats every dataset (all of which contain $n \geq 140{,}000$ samples), as though they contain 100,000 samples:

$$nBIC = \log(100{,}000) \times k - 2 \times (L/n \times 100{,}000)$$

where k is the number of free parameters (k = 1 for the single-exponential model and k = 3 for the bi-exponential model) and L is the likelihood function. This normalization was implemented to maintain methodological consistency with prior work, although all EEG segments were of equal length ($n \approx 140{,}000$ samples) and thus the correction has a negligible effect on the results.

Detrended Fluctuation Analysis (DFA):

We estimated Hurst exponents from band-limited amplitude envelopes of the EEG. Signals were band-pass filtered into 13 logarithmically spaced frequency bins between 1 and 80 Hz using a finite impulse response (FIR) filter designed with the window method (implemented in MNE). Filtering used a Hamming-window FIR filter, applied in zero-phase (bidirectional) mode, with transition bands determined automatically by MNE's default heuristics. Window sizes were initially generated as 81 logarithmically spaced points between 0.1 and 1000 seconds (approximately 20 per decade) and converted to

bioRxiv preprint doi: https://doi.org/10.1101/2025.10.19.683280; this version posted October 20, 2025. The copyright holder for this preprint (which was not certified by peer review) is the author/funder, who has granted bioRxiv a license to display the preprint in perpetuity. It is made available under aCC-BY 4.0 International license.

samples according to the sampling frequency. After rounding to integer samples and restricting the range to the fitting interval [S_{min}, 30 s], approximately 50 distinct window sizes remained for DFA estimation. Windows were slid with 50% overlap. For each frequency bin, the DFA exponent was estimated by fitting a straight line to the log–log plot of fluctuation versus window size within the fitting interval. The upper bound of 30 seconds is conventional in long-range temporal correlation analyses of human resting EEG (Hardstone, Poil et al. 2012, Avramiea, Diachenko et al. 2025). The lower bound S_{min} was band-specific and taken from a predefined schedule [5.0, 5.0, 5.0, 3.981, 3.162, 2.238, 1.412, 1.122, 0.794, 0.562, 0.398, 0.281, 0.141] seconds, matched to the lower edge of each frequency bin.

The DFA computation followed three standard steps:

1. *Signal profile construction*

$$x(t) = \sum_{k=1}^{t} (y(k) - \langle y \rangle)$$

where y(k) is the demeaned signal, ⟨y⟩ denotes the temporal mean of the signal, and x(t) is the integrated profile.

2. *Local detrending and fluctuation*

For each window of length s, a first-order polynomial $y_s(k)$ was fit by ordinary least squares to the profile $Y(k)$ and subtracted. The fluctuation at scale s was then defined as the across-windows average of the within-window standard deviation:

$$F(s) = \langle \sigma(Y(k) - y_s(k)) \rangle$$

bioRxiv preprint doi: https://doi.org/10.1101/2025.10.19.683280; this version posted October 20, 2025. The copyright holder for this preprint (which was not certified by peer review) is the author/funder, who has granted bioRxiv a license to display the preprint in perpetuity. It is made available under aCC-BY 4.0 International license.

3. *Scaling law*

Fluctuations across time scales were assessed by regressing $logF(s)$ against in the fitting interval, yielding the DFA (Hurst) exponent α from the slope:

$$F(s) \propto s^{\alpha}$$

The proportion of high- and low-power oscillations (E/I$_{HLP}$):

The bi-exponential model of the power distribution decomposes the signal into a low-power and a high-power component, which can be interpreted as reflecting inhibitory- and excitatory-dominated network states, respectively. Inhibition stabilizes the system and favors low-amplitude activity, whereas excitation promotes large-amplitude oscillations. Mixing weight δ represents the relative contribution of the two exponential components describing low- and high-power oscillatory states. The model is constrained such that the first exponent corresponds to the low-power state and the second to the high-power state. Thus, δ can be interpreted as the proportion of high-power activity. δ values near 0 indicate predominantly low-power (inhibitory) states, values near 1 indicate predominantly high-power (excitatory) states, and δ values around 0.5 indicate a balanced contribution of both. δ was only interpreted when the signal showed clear bistability (BiS ≥ 2.5) and δ fell within the range 0.02–0.98; otherwise, it was set to NaN.

To ensure interpretability, E/I_{HLP} was only computed when the signal exhibited sufficient bistability (BiS ≥ 2.5) and when δ lay within the range 0.02–0.98; otherwise, E/I_{HLP} values were set to NaN. Under these conditions, δ provides an index of the

bioRxiv preprint doi: https://doi.org/10.1101/2025.10.19.683280; this version posted October 20, 2025. The copyright holder for this preprint (which was not certified by peer review) is the author/funder, who has granted bioRxiv a license to display the preprint in perpetuity. It is made available under aCC-BY 4.0 International license.

proportion of high- to low-power oscillations and serves as a proxy for the excitation–inhibition balance of the underlying network. We applied a threshold of BiS $\geq$ 2.5, under which the δ–BiS relationship becomes stable and δ values closely match independent estimates of excitation–inhibition balance (Avramiea, Diachenko et al. 2025).

Functional E/I ratio (fE/I):

The functional excitation–inhibition ratio (fE/I) provides a model-based estimate of cortical excitation–inhibition balance by quantifying the coupling between oscillatory amplitude and long-range temporal correlations (LRTC) (Bruining, Hardstone et al. 2020). Excitatory activity tends to increase oscillatory amplitude, whereas LRTC increase with excitation up to the critical point and become negatively correlated with excitation beyond that. Thus, the correlation between amplitude and fluctuation scaling provides a proxy for the underlying excitation–inhibition ratio.

1. *Band-pass filtering and amplitude extraction*

EEG signals were first band-pass filtered into the predefined frequency bins using a finite impulse response (FIR) filter (Hamming window, zero-phase, bidirectional). The analytic amplitude Y(t) was then extracted as the absolute value of the Hilbert transform of the filtered signal.

2. *Signal profile construction*

For each channel × frequency bin, the amplitude envelope was demeaned and cumulatively summed to generate a signal profile:

bioRxiv preprint doi: https://doi.org/10.1101/2025.10.19.683280; this version posted October 20, 2025. The copyright holder for this preprint (which was not certified by peer review) is the author/funder, who has granted bioRxiv a license to display the preprint in perpetuity. It is made available under aCC-BY 4.0 International license.

$$x(t) = \sum_{k=1}^{t} (y(k) - \langle y \rangle)$$

where y(k) is the instantaneous amplitude, ⟨y⟩ its temporal mean, and x(t) the integrated profile.

3. *Windowing and amplitude normalization*

The signal profile was segmented into 5-second windows with 80% overlap. Within each window, the profile was divided by the mean amplitude of that window. This normalization step removes scale differences in absolute oscillatory power.

4. *Local detrending*

Within each normalized window, a first-order polynomial $y_S(k)$ was fit by least squares and subtracted from the profile, yielding detrended, amplitude-normalized windows.

5. *Fluctuation function*

For each window, the standard deviation of the detrended, normalized profile was computed to yield the normalized fluctuation function:

$$F(s) = \langle \sigma(X(k) - y_s(k)) \rangle$$

6. *Correlation with amplitude*

Across windows, we computed the Pearson correlation coefficient r between the windowed mean amplitude and the windowed normalized fluctuation F(s). The fE/I ratio was then defined as:

$$fE/I = 1 - r(W_{amp}, W_{F(s)})$$

Where W_{amp} denotes windowed mean amplitudes and $W_{F(s)}$ the corresponding

bioRxiv preprint doi: https://doi.org/10.1101/2025.10.19.683280; this version posted October 20, 2025. The copyright holder for this preprint (which was not certified by peer review) is the author/funder, who has granted bioRxiv a license to display the preprint in perpetuity. It is made available under aCC-BY 4.0 International license.

windowed fluctuations. By construction, $fE/I \approx 1$ indicates balanced excitation–inhibition dynamics, whereas values above or below 1 reflect shifts toward excitation- or inhibition-dominated regimes, respectively.

7. Quality control

Following prior work, the fE/I estimate was set to NaN when the DFA exponent of the underlying signal profile did not exceed a minimum threshold of 0.6, ensuring that fE/I was only computed in signals with sufficient long-range temporal correlations.

Excitation-inhibition strength

Separation of High- and Low- Power Oscillations (E+I_{HLS}):

We quantified how separated high- and low-amplitude states were in the power distribution – an index of combined excitatory and inhibitory strength (E+I; Avramiea, Diachenko et al., 2025). For each bi-exponential fit, we identified the location of the low-power and high-power peaks and computed their distance on a logarithmic scale:

$$E + I_{HLS} = \log_{10}(H) - \log_{10}(L)$$

Where L and H denote the positions of the low- and high-power peaks, respectively. This measure reflects the order-of-magnitude gap between the two states, with larger values indicating a clearer distinction between low- and high-amplitude oscillatory regimes.

To ensure reliable interpretation, E+I_{HLS} was only calculated when the signal showed sufficient bistability (BiS ≥ 2.5) and when both states contributed substantially to the distribution ($0.02 \leq \delta \leq 0.98$). In all other cases, E+I_{HLS} values were set to NaN.

bioRxiv preprint doi: https://doi.org/10.1101/2025.10.19.683280; this version posted October 20, 2025. The copyright holder for this preprint (which was not certified by peer review) is the author/funder, who has granted bioRxiv a license to display the preprint in perpetuity. It is made available under aCC-BY 4.0 International license.

Lempel–Ziv complexity (LZC):

We estimated signal irregularity by computing Lempel–Ziv complexity (LZC) using a single-pass Lempel–Ziv–Welch (LZW) dictionary-building procedure in MATLAB 2020a (Comsa 2019). Starting from an initial dictionary containing the two symbols [0, 1] the binary sequence was scanned left-to-right. At each step, the current phrase was extended by one symbol; when the extended phrase was not found in the dictionary, it was added as a new entry, and a new phrase was started. The complexity of the sequence was defined as the final dictionary size, representing the number of unique patterns identified. To account for sequence length and symbol frequency, we normalized this value by dividing it by the mean complexity obtained from ten randomly shuffled versions of the same sequence (N=10). Larger normalized values indicate more irregular or less structured signals. We note that the implementation uses random shuffling without an explicitly fixed seed; thus, normalized values may vary minimally across runs.

Although the ~4.65-min time series was assembled from five separate segments (49–60–60–60–49 s) rather than a single continuous recording, this does not compromise the reliability of the LZC estimates. Rivolta et al. demonstrated that as few as 1,000 datapoints are sufficient for stable LZC estimation during sleep (Rivolta, Migliorini et al. 2014), and each of our segments greatly exceeded this length.

Statistical analysis for BiS, DFA, fE/I, E/I$_{HLP}$, and E+I$_{HLS}$

For E/I$_{HLP}$ and E+I$_{HLS}$, channel-level estimates returned some NaN’s as described above.

bioRxiv preprint doi: https://doi.org/10.1101/2025.10.19.683280; this version posted October 20, 2025. The copyright holder for this preprint (which was not certified by peer review) is the author/funder, who has granted bioRxiv a license to display the preprint in perpetuity. It is made available under aCC-BY 4.0 International license.

At the subject level, we averaged across channels, omitting NaNs, such that values were computed from available channels only. If all channels were missing for a given subject and band, the subject was excluded from subsequent group-level analyses.

Machine learning classification with LASSO

We trained a LASSO-regularized logistic regression classifier to distinguish MDD from HC. The feature matrix comprised EEG-derived biomarkers indexing criticality (BiS), excitation–inhibition balance (E/I_{HLP}, fE/I), excitation-inhibition strength ($E+I_{HLS}$), and complexity (LZC), with age and sex added as covariates. To minimize contamination by potential high-γ artifacts, we excluded bands 11, 12, and 13 (35.2-44.8 Hz, 44.8-57.1 Hz, and 57.1-72.7 Hz, respectively) for BiS, fE/I, E/I_{HLP}, and $E+I_{HLS}$. Rows containing missing (NaN) or non-finite (Inf) values were excluded. Participants (183 MDD, 133 HC) were split into stratified training (70%) and test (30%) sets with a fixed random seed, ensuring balanced class ratios. Features were standardized to zero mean and unit variance using training-set statistics, with the same parameters applied to test data to prevent data leakage.

For the classifier, 100 candidate regularization parameters (λ) were evaluated by 10-fold cross-validation within the training set. The optimal λ was selected according to the one-standard-error rule, yielding a sparse and interpretable model. Non-zero coefficients at this λ were retained as predictive features, and an unregularized logistic regression model was refit using only these features on the training data and then applied

bioRxiv preprint doi: https://doi.org/10.1101/2025.10.19.683280; this version posted October 20, 2025. The copyright holder for this preprint (which was not certified by peer review) is the author/funder, who has granted bioRxiv a license to display the preprint in perpetuity. It is made available under aCC-BY 4.0 International license.

to the held-out test set. Predicted probabilities of MDD were converted to class labels (MDD if ≥0.5, HC if <0.5). Model performance was quantified by area under the receiver operating characteristic curve (AUC), accuracy, sensitivity, and specificity.

Figure 5A illustrates (a) non-zero model coefficients, (b) the number of features selected across λ values (regularization path), and (c) ROC curves for training and test sets.

We also evaluated models including DFA and γ-band features. Because our EEG data lengths were shorter than the traditional length recommended for reliable DFA estimation, and γ-band activity may partly reflect muscle noise, these measures were not included in the main analysis (Figure 5A). For completeness, Supplemental Figure 3A shows classification performance when DFA was added, and Supplemental Information reports results from models including both DFA and γ-band features.

bioRxiv preprint doi: https://doi.org/10.1101/2025.10.19.683280; this version posted October 20, 2025. The copyright holder for this preprint (which was not certified by peer review) is the author/funder, who has granted bioRxiv a license to display the preprint in perpetuity. It is made available under aCC-BY 4.0 International license.

Supplemental Results

LASSO classification with DFA

We next examined whether adding DFA features altered classifier performance. At the optimal λ (10-fold CV, 1SE rule), the model retained features, including markers of criticality (BiS, DFA), E–I balance (fE/I, E/I_{HLP}), E–I strength ($E+I_{HLS}$), age, and sex. The coefficient map showed the largest positive weights on E/I_{HLP} (hlp b 02 (θ: 4.0-5.1 Hz) = +0.194, hlp b 03 (θ: 3.1-6.5 Hz) = +0.121) and fE/I (fei b 02 (θ: 4.0-5.1 Hz) = +0.133), whereas the largest negative weights were on fE/I (fei b 01 (δ: 1.0-4.0 Hz) = −0.382), $E+I_{HLS}$ (hls b 05 (α: 8.3-10.5 Hz) = −0.193, hls b 08 (β: 17.0-21.7 Hz) = −0.121), BiS (bis b 07 (β: 13.4-17.0 Hz) = −0.182, bis b 05 (α: 8.3-10.5 Hz) = −0.065), and DFA (dfa b 05 (α : 8.3-10.5 Hz) = −0.128, dfa b 06 (α: 10.5-13.4 Hz) = −0.009) (Supp. Fig. 5Aa). The path plot illustrates progressive sparsity as λ increases, with the number of non-zero coefficients steadily decreasing (Supp. Fig. 3Ab). At the optimal λ determined by cross-validation, the model retained 13 features. ROC curves indicated above-chance, but weaker discrimination compared with the DFA-excluded model: training AUC = 0.84 (ACC = 0.76), test AUC = 0.75 (ACC = 0.68, sensitivity = 0.77, specificity = 0.56) (Supp. Fig. 3Ac).

Together, these results show that while DFA features were selected by the model, their inclusion did not improve generalization, and test performance remained lower than the model without DFA.

bioRxiv preprint doi: https://doi.org/10.1101/2025.10.19.683280; this version posted October 20, 2025. The copyright holder for this preprint (which was not certified by peer review) is the author/funder, who has granted bioRxiv a license to display the preprint in perpetuity. It is made available under aCC-BY 4.0 International license.

LASSO classification with full γ-bands

Including γ-band features (bands 11–13) yielded a LASSO model that retained 12 predictors, with the coefficient map (Supp. Fig. 3Ba) dominated by large γ-band BiS and E+I_{HLS} weights (bis b 12 (γ: 44.8-57.1 Hz) = −2.42, hls b 13 (γ: 57.1-72.7 Hz) = +0.80). The path plot showed a sharp drop in the number of selected coefficients near λ ≈ 0, followed by a more gradual decline as λ increased (Supp. Fig. 3Bb). At the cross-validated optimal λ, 12 predictors were retained. The ROC curve (Supp. Fig. 3Bc) indicated excellent performance (train AUC = 0.99, test AUC = 0.95; ACC = 0.90, sensitivity = 0.90, specificity = 0.90). However, the coefficient map (Supp. Fig. 3Ba) was dominated by γ-band features, with BiS in band 12 showing the largest weight (β = −2.42). These features likely contributed substantially to the improved discrimination. However, because high-frequency EEG is particularly susceptible to muscle artifacts, the γ-inclusive findings should be interpreted with caution and are reported only as supplemental.

bioRxiv preprint doi: https://doi.org/10.1101/2025.10.19.683280; this version posted October 20, 2025. The copyright holder for this preprint (which was not certified by peer review) is the author/funder, who has granted bioRxiv a license to display the preprint in perpetuity. It is made available under aCC-BY 4.0 International license.

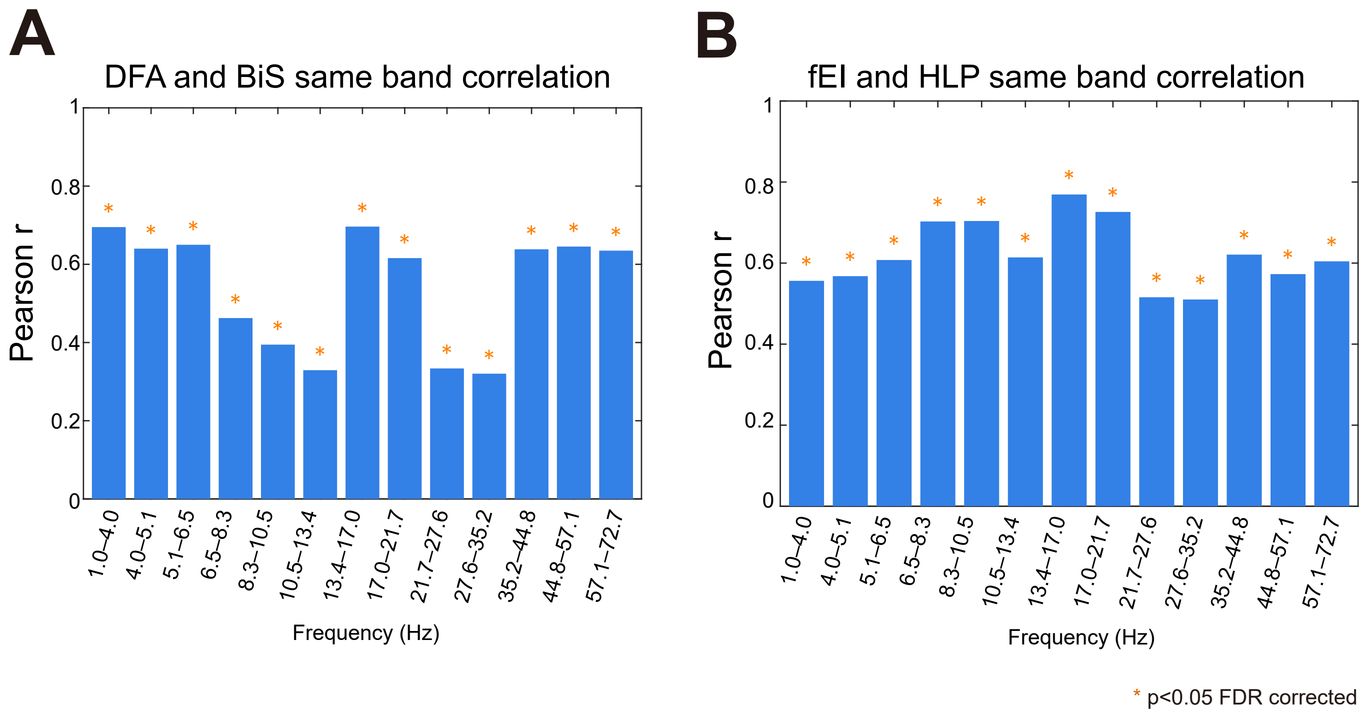

Supplemental Figure 1

Supplemental Figure 1. Same-band correlations between metrics.

(A) Pearson correlation coefficients (r) between detrended fluctuation analysis (DFA) and bistability (BiS) across frequency bands. (B) Pearson correlation coefficients (r) between functional excitation–inhibition ratio (fEI) and high-to-low power ratio (HLP) across frequency bands. Orange asterisks indicate statistical significance (* $p < 0.05$, FDR corrected for 13 frequency bands).

All available participants ($n = 316$) were included in the analyses, with missing values treated as NaNs and skipped in pairwise correlations.

bioRxiv preprint doi: https://doi.org/10.1101/2025.10.19.683280; this version posted October 20, 2025. The copyright holder for this preprint (which was not certified by peer review) is the author/funder, who has granted bioRxiv a license to display the preprint in perpetuity. It is made available under aCC-BY 4.0 International license.

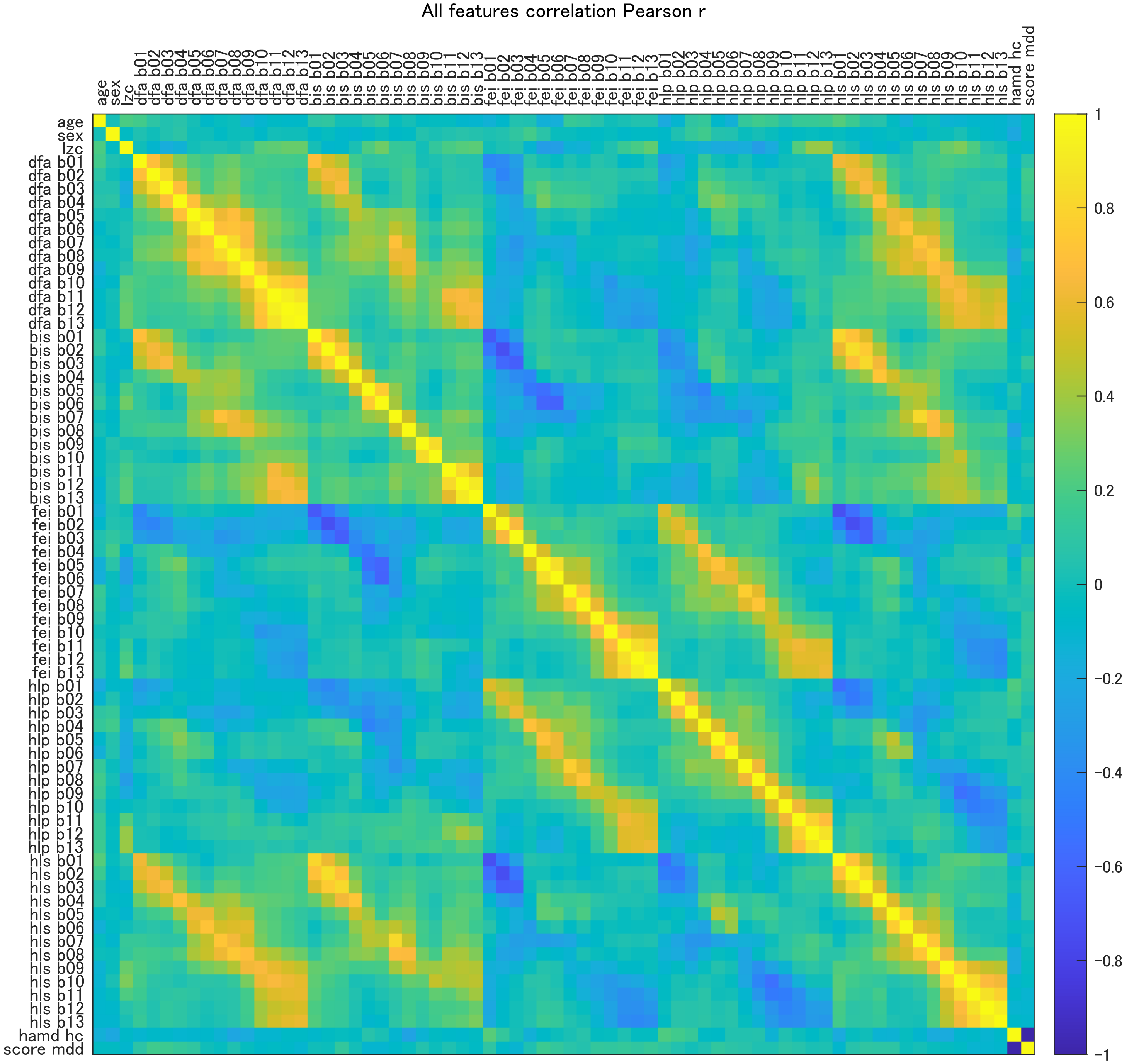

Supplemental Figure 2

Supplemental Figure 2. Feature-to-feature correlation matrix.
Values represent the Pearson correlation coefficient between pairs of extracted features, age, sex, and depression scores (HAMD for HC ≤(mamd hc), IDS-SR for MDD (mdd score)). The right color scale indicates the correlation strength ($-1 \leq r \leq 1$). Warm colors (yellow) indicate positive correlations, and cool colors (blue) indicate negative correlations. Frequency bands b01–b13 correspond to 13 logarithmically spaced sub-bands.

bioRxiv preprint doi: https://doi.org/10.1101/2025.10.19.683280; this version posted October 20, 2025. The copyright holder for this preprint (which was not certified by peer review) is the author/funder, who has granted bioRxiv a license to display the preprint in perpetuity. It is made available under aCC-BY 4.0 International license.

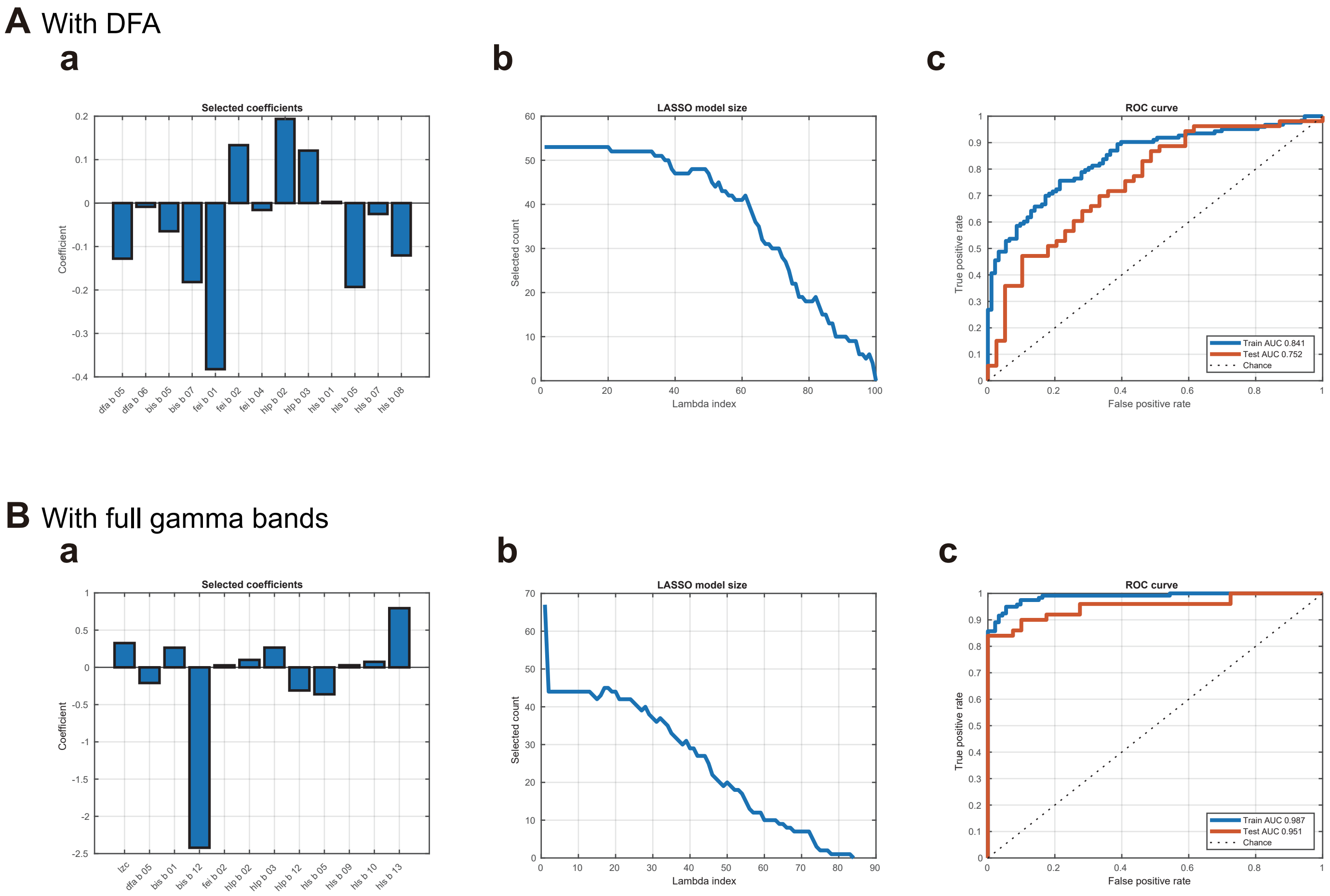

Supplemental Figure 3

Supplemental Figure 3. Machine learning classification with and without DFA and full gamma bands.

(A) With DFA included. (Aa) LASSO logistic regression coefficients for the optimal model. Frequency bands b01–b13 correspond to 13 logarithmically spaced sub-bands. (Ab) Path plot indicates model sparsity across λ values (model size as a function of the regularization parameter). (Ac) Receiver operating characteristic (ROC) curves demonstrate classification performance in the training and test sets (Train AUC = 0.841, Test AUC = 0.752). (B) With full gamma bands included. (Ba) LASSO coefficients for the optimal model reveal additional contributions from high-γ features. (Bb) Path plot indicates sparsity across λ values. (Bc) ROC curves show improved discrimination (Train AUC = 0.987, Test AUC = 0.951).

bioRxiv preprint doi: https://doi.org/10.1101/2025.10.19.683280; this version posted October 20, 2025. The copyright holder for this preprint (which was not certified by peer review) is the author/funder, who has granted bioRxiv a license to display the preprint in perpetuity. It is made available under aCC-BY 4.0 International license.

	N	Age Mean (years)	Age SD	Male (Female)	Male/Female	HAM-D score	HAM-D SD	IDS-SR score	IDS-SR SD
HC	133	44.91	21.36	55 (78)	0.41	2.54	2.61		
MDD	183	46.75	16.22	60 (123)	0.33			46.67	10.33

Supplemental Table 1

Supplemental Table 1.

Demographics and clinical scores for healthy controls (HC) and patients with major depressive disorder (MDD). Values are mean ± SD unless noted. Sample sizes: HC N=133; MDD N=183. Age: HC 44.91 ± 21.36 years (HC age was recorded in 5-year bins; mean and SD were estimated from bin midpoints); MDD 46.75 ± 16.22 years; between-group difference not significant (Welch $t = -0.84$, $p = 0.404$; 95% CI −6.19 to 2.50). Sex: HC 55 male and 78 female (male/female ratio 0.41); MDD 60 male and 123 female (male/female ratio 0.33); distribution not significantly different between groups (chi-square = 2.44, df = 1, $p = 0.118$; Cramer's V = 0.088). Depression measures are group-specific and cannot be compared across groups: HC, Hamilton Depression Rating Scale 2.54 ± 2.61; MDD, Inventory of Depressive Symptomatology Self-Report 46.67 ± 10.33.

				HC			MDD							
Band	Lower (Hz)	Upper (Hz)	Freq. band	n	Mean	SE	n	Mean	SE	cohen d	CI (low)	CI (high)	p-value	p (FDR)
1	1.0	4.0	delta	133	3.414	0.031	183	3.466	0.027	-0.142	-0.366	0.081	0.210	0.210
2	4.0	5.1	theta	133	3.561	0.027	183	3.439	0.030	0.328	0.103	0.552	0.003	0.004
3	5.1	6.5	theta	133	3.639	0.029	183	3.526	0.028	0.310	0.086	0.535	0.006	0.008
4	6.5	8.3	theta	133	3.845	0.036	183	3.575	0.049	0.469	0.243	0.695	0.000	0.000
5	8.3	10.5	alpha	133	3.764	0.071	183	3.195	0.084	0.563	0.336	0.791	0.000	0.000
6	10.5	13.4	alpha	133	3.614	0.079	183	3.172	0.079	0.440	0.214	0.666	0.000	0.000
7	13.4	17.0	beta	133	3.684	0.022	183	3.496	0.019	0.735	0.504	0.965	0.000	0.000
8	17.0	21.7	beta	133	3.588	0.020	183	3.481	0.017	0.457	0.231	0.683	0.000	0.000
9	21.7	27.6	beta	133	3.522	0.019	183	3.410	0.036	0.279	0.055	0.504	0.007	0.008
10	27.6	35.2	gamma	133	3.533	0.023	183	3.425	0.040	0.244	0.020	0.469	0.019	0.020
11	35.2	44.8	gamma	133	3.601	0.027	183	2.678	0.082	1.062	0.824	1.301	0.000	0.000
12	44.8	57.1	gamma	133	3.660	0.029	183	2.172	0.085	1.642	1.384	1.899	0.000	0.000
13	57.1	72.7	gamma	133	3.736	0.031	183	2.654	0.077	1.311	1.066	1.557	0.000	0.000

Supplementary Table 2. Unadjusted group comparison statistics: Bistability (BiS).

Summary statistics from the unadjusted comparison between major depressive disorder (MDD) and healthy controls (HC). For each subject, metric values were averaged across channels. Group-level comparisons between MDD and HC were performed separately for each frequency band using Welch's unequal-variance t-tests. Resulting p-values were adjusted for multiple testing across the 13 frequency bands within each metric using the Benjamini–Hochberg false discovery rate (FDR) procedure. Reported statistics include group means (±SE) for HC and MDD, effect sizes as Cohen's d (MDD − HC), and corresponding 95% confidence intervals for d. n indicates the number of subjects included in each group for a given frequency band. Abbreviations: HC, healthy controls; MDD, major depressive disorder; SE, standard error; CI, confidence interval; FDR, false discovery rate; Freq. Band, frequency band.

				HC			MDD							
Band	Lower (Hz)	Upper (Hz)	Freq. band	n	Mean	SE	n	Mean	SE	cohen d	CI (low)	CI (high)	p-value	p (FDR)
1	1.0	4.0	delta	133	0.610	0.005	183	0.613	0.004	-0.049	-0.272	0.175	0.669	0.669
2	4.0	5.1	theta	133	0.602	0.005	183	0.599	0.004	0.050	-0.174	0.273	0.659	0.669
3	5.1	6.5	theta	133	0.619	0.006	183	0.615	0.005	0.053	-0.170	0.276	0.640	0.669
4	6.5	8.3	theta	133	0.686	0.009	183	0.659	0.007	0.283	0.058	0.507	0.014	0.019
5	8.3	10.5	alpha	133	0.741	0.008	183	0.680	0.007	0.625	0.396	0.853	0.000	0.000
6	10.5	13.4	alpha	133	0.739	0.008	183	0.676	0.007	0.693	0.464	0.923	0.000	0.000
7	13.4	17.0	beta	133	0.682	0.006	183	0.649	0.005	0.511	0.284	0.737	0.000	0.000
8	17.0	21.7	beta	133	0.676	0.005	183	0.638	0.004	0.657	0.428	0.886	0.000	0.000
9	21.7	27.6	beta	133	0.665	0.004	183	0.634	0.004	0.614	0.386	0.842	0.000	0.000
10	27.6	35.2	gamma	133	0.649	0.004	183	0.634	0.003	0.337	0.112	0.562	0.003	0.005
11	35.2	44.8	gamma	133	0.658	0.005	183	0.628	0.004	0.535	0.308	0.762	0.000	0.000
12	44.8	57.1	gamma	133	0.672	0.005	183	0.643	0.005	0.481	0.254	0.707	0.000	0.000
13	57.1	72.7	gamma	133	0.686	0.005	183	0.665	0.005	0.331	0.106	0.556	0.003	0.005

Supplementary Table 3. Unadjusted group comparison statistics: Detrended fluctuation analysis (DFA).
Statistical analysis identical to that described for Supplementary Table 2 (BiS). Reported statistics include group means (±SE) for HC and MDD, effect sizes as Cohen's d (MDD − HC), 95% confidence intervals for d, and p- and FDR-adjusted p-values.

				HC			MDD							
Band	Lower (Hz)	Upper (Hz)	Freq. band	n	Mean	SE	n	Mean	SE	cohen d	CI (low)	CI (high)	p-value	p (FDR)
1	1.0	4.0	delta	133	0.254	0.008	183	0.261	0.007	-0.068	-0.292	0.155	0.548	0.647
2	4.0	5.1	theta	133	0.293	0.009	183	0.340	0.009	-0.421	-0.646	-0.195	0.000	0.001
3	5.1	6.5	theta	133	0.299	0.008	183	0.358	0.009	-0.526	-0.753	-0.299	0.000	0.000
4	6.5	8.3	theta	133	0.378	0.013	182	0.412	0.011	-0.222	-0.446	0.002	0.053	0.087
5	8.3	10.5	alpha	132	0.505	0.017	182	0.501	0.013	0.020	-0.204	0.244	0.866	0.919
6	10.5	13.4	alpha	133	0.497	0.016	180	0.495	0.013	0.012	-0.212	0.236	0.919	0.919
7	13.4	17.0	beta	133	0.428	0.012	183	0.460	0.009	-0.248	-0.472	-0.024	0.032	0.059
8	17.0	21.7	beta	133	0.462	0.011	183	0.477	0.009	-0.120	-0.343	0.104	0.300	0.434
9	21.7	27.6	beta	133	0.430	0.009	180	0.418	0.010	0.095	-0.129	0.319	0.390	0.508
10	27.6	35.2	gamma	133	0.337	0.009	180	0.308	0.009	0.254	0.029	0.479	0.024	0.052
11	35.2	44.8	gamma	133	0.288	0.009	177	0.201	0.009	0.765	0.532	0.998	0.000	0.000
12	44.8	57.1	gamma	133	0.264	0.010	177	0.158	0.008	0.995	0.757	1.233	0.000	0.000
13	57.1	72.7	gamma	133	0.252	0.010	181	0.182	0.008	0.634	0.404	0.863	0.000	0.000

Supplementary Table 4. Unadjusted group comparison statistics: High-to-low power ratio (E/I_{HLP}).

Statistical analysis identical to that described for Supplementary Table 2 (BiS). Reported statistics include group means (±SE) for HC and MDD, effect sizes as Cohen's d (MDD − HC), 95% confidence intervals for d, and p- and FDR-adjusted p-values.

				HC			MDD							
Band	Lower (Hz)	Upper (Hz)	Freq. band	n	Mean	SE	n	Mean	SE	cohen d	CI (low)	CI (high)	p-value	p (FDR)
1	1.0	4.0	delta	133	0.864	0.008	183	0.840	0.007	0.253	0.029	0.477	0.027	0.087
2	4.0	5.1	theta	133	0.897	0.008	183	0.935	0.008	-0.376	-0.601	-0.151	0.001	0.009
3	5.1	6.5	theta	133	0.896	0.008	183	0.929	0.008	-0.313	-0.538	-0.089	0.005	0.030
4	6.5	8.3	theta	133	0.921	0.013	182	0.928	0.011	-0.043	-0.267	0.180	0.706	0.809
5	8.3	10.5	alpha	133	1.023	0.021	183	1.035	0.014	-0.059	-0.282	0.165	0.622	0.809
6	10.5	13.4	alpha	133	0.977	0.017	183	1.001	0.012	-0.134	-0.358	0.089	0.252	0.547
7	13.4	17.0	beta	133	0.937	0.011	183	0.949	0.008	-0.102	-0.325	0.122	0.387	0.719
8	17.0	21.7	beta	133	0.959	0.010	183	0.964	0.007	-0.046	-0.269	0.178	0.695	0.809
9	21.7	27.6	beta	133	0.928	0.008	183	0.930	0.007	-0.028	-0.251	0.196	0.809	0.809
10	27.6	35.2	gamma	133	0.900	0.009	183	0.897	0.008	0.028	-0.195	0.251	0.806	0.809
11	35.2	44.8	gamma	133	0.867	0.011	182	0.832	0.010	0.275	0.051	0.500	0.016	0.070
12	44.8	57.1	gamma	133	0.846	0.013	183	0.813	0.011	0.220	-0.004	0.444	0.054	0.140
13	57.1	72.7	gamma	133	0.820	0.014	183	0.809	0.012	0.068	-0.156	0.291	0.550	0.809

Supplementary Table 5. Unadjusted group comparison statistics: functional E/I ratio (fE/I).

Statistical analysis identical to that described for Supplementary Table 2 (BiS). Reported statistics include group means (±SE) for HC and MDD, effect sizes as Cohen’s d (MDD − HC), 95% confidence intervals for d, and p- and FDR-adjusted p-values.

				HC			MDD							
Band	Lower (Hz)	Upper (Hz)	Freq. band	n	Mean	SE	n	Mean	SE	cohen d	CI (low)	CI (high)	p-value	p (FDR)
1	1.0	4.0	delta	133	0.493	0.009	183	0.517	0.008	-0.215	-0.438	0.009	0.058	0.083
2	4.0	5.1	theta	133	0.498	0.008	183	0.480	0.008	0.165	-0.059	0.388	0.138	0.180
3	5.1	6.5	theta	133	0.507	0.009	183	0.490	0.008	0.162	-0.061	0.386	0.153	0.180
4	6.5	8.3	theta	133	0.609	0.014	182	0.548	0.010	0.410	0.184	0.636	0.000	0.001
5	8.3	10.5	alpha	132	0.760	0.020	182	0.606	0.017	0.657	0.427	0.887	0.000	0.000
6	10.5	13.4	alpha	133	0.666	0.017	180	0.542	0.014	0.638	0.408	0.867	0.000	0.000
7	13.4	17.0	beta	133	0.509	0.008	183	0.444	0.006	0.762	0.531	0.993	0.000	0.000
8	17.0	21.7	beta	133	0.479	0.006	183	0.424	0.006	0.729	0.499	0.960	0.000	0.000
9	21.7	27.6	beta	133	0.431	0.007	180	0.407	0.006	0.306	0.080	0.531	0.008	0.013
10	27.6	35.2	gamma	133	0.440	0.009	180	0.434	0.008	0.059	-0.166	0.283	0.605	0.605
11	35.2	44.8	gamma	133	0.489	0.010	177	0.506	0.010	-0.138	-0.363	0.088	0.226	0.245
12	44.8	57.1	gamma	133	0.536	0.011	177	0.578	0.010	-0.311	-0.538	-0.085	0.006	0.013
13	57.1	72.7	gamma	133	0.571	0.011	181	0.614	0.011	-0.298	-0.523	-0.073	0.008	0.013

Supplementary Table 6. Unadjusted group comparison statistics: Separation of high- and low-power oscillations ($E+I_{HLS}$).

Statistical analysis identical to that described for Supplementary Table 2 (BiS). Reported statistics include group means (±SE) for HC and MDD, effect sizes as Cohen’s d (MDD − HC), 95% confidence intervals for d, and p- and FDR-adjusted p-values.

				HC			MDD							
Band	Lower (Hz)	Upper (Hz)	Freq. band	n	Mean	SE	n	Mean	SE	cohen d	CI (low)	CI (high)	p-value	p (FDR)
1	1.0	4.0	delta	133	-0.023	0.026	183	0.017	0.022	-0.077	0.369	0.145	0.201	0.238
2	4.0	5.1	theta	133	0.052	0.022	183	-0.039	0.021	-0.603	-0.150	-0.374	0.001	0.002
3	5.1	6.5	theta	133	0.049	0.026	183	-0.035	0.022	-0.528	-0.078	-0.301	0.008	0.011
4	6.5	8.3	theta	133	0.082	0.032	183	-0.063	0.028	-0.646	-0.191	-0.416	0.000	0.000
5	8.3	10.5	alpha	133	0.134	0.045	183	-0.111	0.041	-0.727	-0.268	-0.495	0.000	0.000
6	10.5	13.4	alpha	133	0.134	0.039	183	-0.105	0.032	-0.831	-0.366	-0.595	0.000	0.000
7	13.4	17.0	beta	133	0.088	0.019	183	-0.063	0.016	-1.003	-0.527	-0.760	0.000	0.000
8	17.0	21.7	beta	133	0.038	0.016	183	-0.026	0.012	-0.647	-0.192	-0.417	0.000	0.000
9	21.7	27.6	beta	133	0.005	0.016	183	-0.004	0.013	-0.281	0.165	-0.057	0.613	0.664
10	27.6	35.2	gamma	133	0.005	0.017	183	-0.004	0.015	-0.265	0.181	-0.042	0.712	0.712
11	35.2	44.8	gamma	133	0.118	0.024	183	-0.127	0.039	-0.870	-0.403	-0.632	0.000	0.000
12	44.8	57.1	gamma	133	0.767	0.032	183	-0.591	0.082	-1.999	-1.415	-1.696	0.000	0.000
13	57.1	72.7	gamma	133	0.516	0.031	183	-0.411	0.072	-1.501	-0.978	-1.232	0.000	0.000

Supplementary Table 7. Covariate-adjusted group comparison statistics: Bistability (BiS).

Summary statistics from the covariate-adjusted comparison between major depressive disorder (MDD) and healthy controls (HC). For each subject, metric values were averaged across channels. Group-level analyses were performed separately for each frequency band using robust linear regression models with group (MDD vs. HC) as the predictor of interest and age (z-scored) and sex (male = 1, female = 0) as covariates. To obtain covariate-adjusted group differences, group coefficients and their corresponding t-statistics were extracted from the full model. Reported statistics include covariate-adjusted group means (±SE) for HC and MDD, effect sizes as Cohen's d (MDD − HC), and corresponding 95% confidence intervals for d. n indicates the number of subjects included in each group for a given frequency band. Resulting p-values were adjusted for multiple testing across the 13 frequency bands within each metric using the Benjamini–Hochberg false discovery rate (FDR) procedure. Abbreviations: HC, healthy controls; MDD, major depressive disorder; SE, standard error; CI, confidence interval; FDR, false discovery rate; Freq. Band, frequency band.

				HC			MDD							
Band	Lower (Hz)	Upper (Hz)	Freq. band	n	Mean	SE	n	Mean	SE	cohen d	CI (low)	CI (high)	p-value	p (FDR)
1	1.0	4.0	delta	133	0.007	0.005	183	0.010	0.004	0.045	-0.009	0.014	0.417	0.493
2	4.0	5.1	theta	133	0.009	0.005	183	0.006	0.004	-0.057	-0.016	0.010	0.589	0.589
3	5.1	6.5	theta	133	0.009	0.006	183	0.006	0.005	-0.052	-0.019	0.012	0.554	0.589
4	6.5	8.3	theta	133	0.024	0.008	183	-0.002	0.007	-0.270	-0.047	-0.004	0.014	0.019
5	8.3	10.5	alpha	133	0.044	0.008	183	-0.017	0.007	-0.617	-0.082	-0.039	0.000	0.001
6	10.5	13.4	alpha	133	0.044	0.008	183	-0.017	0.007	-0.679	-0.081	-0.041	0.000	0.001
7	13.4	17.0	beta	133	0.028	0.006	183	-0.006	0.005	-0.515	-0.047	-0.019	0.000	0.001
8	17.0	21.7	beta	133	0.031	0.005	183	-0.008	0.004	-0.658	-0.052	-0.026	0.000	0.001
9	21.7	27.6	beta	133	0.024	0.004	183	-0.006	0.004	-0.612	-0.041	-0.019	0.000	0.001
10	27.6	35.2	gamma	133	0.015	0.004	183	-0.001	0.003	-0.340	-0.026	-0.005	0.003	0.004
11	35.2	44.8	gamma	133	0.023	0.005	183	-0.006	0.004	-0.528	-0.041	-0.017	0.000	0.001
12	44.8	57.1	gamma	133	0.025	0.005	183	-0.003	0.005	-0.472	-0.042	-0.015	0.000	0.001
13	57.1	72.7	gamma	133	0.021	0.005	183	0.000	0.005	-0.323	-0.035	-0.007	0.000	0.001

Supplementary Table 8. Covariate-adjusted group comparison statistics: Detrended fluctuation analysis (DFA).

Statistical analysis was identical to that described for Supplementary Table 7 (BiS). Reported statistics include group means (±SE) for HC and MDD, effect sizes as Cohen’s *d* (MDD − HC), 95% confidence intervals for *d*, and *p*- and FDR-adjusted *p*-values.

				HC			MDD							
Band	Lower (Hz)	Upper (Hz)	Freq. band	n	Mean	SE	n	Mean	SE	cohen d	CI (low)	CI (high)	p-value	p (FDR)
1	1.0	4.0	delta	133	-0.003	0.007	183	0.002	0.006	-0.162	0.284	0.061	0.591	0.698
2	4.0	5.1	theta	133	-0.025	0.008	183	0.019	0.008	0.214	0.670	0.440	0.000	0.000
3	5.1	6.5	theta	133	-0.029	0.008	183	0.022	0.008	0.299	0.759	0.526	0.000	0.000
4	6.5	8.3	theta	133	-0.020	0.012	182	0.014	0.010	0.033	0.483	0.257	0.024	0.045
5	8.3	10.5	alpha	132	-0.001	0.016	182	0.001	0.013	-0.214	0.233	0.009	0.935	0.950
6	10.5	13.4	alpha	133	-0.001	0.015	180	0.000	0.012	-0.217	0.231	0.007	0.950	0.950
7	13.4	17.0	beta	133	-0.015	0.010	183	0.011	0.009	0.020	0.468	0.243	0.033	0.054
8	17.0	21.7	beta	133	-0.006	0.010	183	0.004	0.008	-0.127	0.319	0.095	0.401	0.521
9	21.7	27.6	beta	133	0.007	0.008	180	-0.006	0.009	-0.353	0.095	-0.128	0.261	0.377
10	27.6	35.2	gamma	133	0.016	0.009	180	-0.012	0.008	-0.511	-0.059	-0.284	0.013	0.029
11	35.2	44.8	gamma	133	0.048	0.008	177	-0.036	0.008	-1.162	-0.668	-0.909	0.000	0.000
12	44.8	57.1	gamma	133	0.054	0.008	177	-0.039	0.007	-1.341	-0.830	-1.079	0.000	0.000
13	57.1	72.7	gamma	133	0.037	0.009	181	-0.026	0.007	-0.924	-0.451	-0.683	0.000	0.000

Supplementary Table 9. Covariate-adjusted group comparison statistics: High-to-low power ratio (E/I_{HLP}).

Statistical analysis was identical to that described for Supplementary Table 7 (BiS). Reported statistics include group means (±SE) for HC and MDD, effect sizes as Cohen's *d* (MDD − HC), 95% confidence intervals for *d*, and *p*- and FDR-adjusted *p*-values.

				HC			MDD							
Band	Lower (Hz)	Upper (Hz)	Freq. band	n	Mean	SE	n	Mean	SE	cohen d	CI (low)	CI (high)	p-value	p (FDR)
1	1.0	4.0	delta	133	0.010	0.007	183	-0.007	0.006	-0.449	-0.001	-0.223	0.049	0.128
2	4.0	5.1	theta	133	-0.025	0.007	183	0.019	0.006	0.394	0.861	0.624	0.000	0.000
3	5.1	6.5	theta	133	-0.020	0.007	183	0.015	0.007	0.240	0.697	0.465	0.000	0.000
4	6.5	8.3	theta	133	-0.006	0.011	182	0.004	0.009	-0.133	0.313	0.089	0.431	0.497
5	8.3	10.5	alpha	133	-0.010	0.018	183	0.007	0.012	-0.122	0.324	0.101	0.375	0.497
6	10.5	13.4	alpha	133	-0.014	0.014	183	0.010	0.011	-0.046	0.401	0.176	0.120	0.261
7	13.4	17.0	beta	133	-0.005	0.010	183	0.004	0.007	-0.118	0.328	0.104	0.359	0.497
8	17.0	21.7	beta	133	-0.001	0.009	183	0.000	0.006	-0.212	0.234	0.011	0.923	0.923
9	21.7	27.6	beta	133	-0.004	0.007	183	0.003	0.006	-0.125	0.321	0.098	0.390	0.497
10	27.6	35.2	gamma	133	-0.004	0.007	183	0.003	0.006	-0.138	0.307	0.084	0.459	0.497
11	35.2	44.8	gamma	133	0.019	0.009	182	-0.014	0.008	-0.550	-0.098	-0.322	0.005	0.021
12	44.8	57.1	gamma	133	0.019	0.011	183	-0.014	0.010	-0.493	-0.043	-0.266	0.019	0.062
13	57.1	72.7	gamma	133	0.008	0.013	183	-0.006	0.011	-0.328	0.118	-0.104	0.358	0.497

Supplementary Table 10. Covariate-adjusted group comparison statistics: functional E/I ratio (fE/I).

Statistical analysis was identical to that described for Supplementary Table 7 (BiS). Reported statistics include group means (±SE) for HC and MDD, effect sizes as Cohen's *d* (MDD − HC), 95% confidence intervals for *d*, and *p*- and FDR-adjusted *p*-values.

				HC			MDD							
Band	Lower (Hz)	Upper (Hz)	Freq. band	n	Mean	SE	n	Mean	SE	cohen d	CI (low)	CI (high)	p-value	p (FDR)
1	1.0	4.0	delta	133	-0.010	0.007	183	0.008	0.006	0.025	0.474	0.248	0.029	0.038
2	4.0	5.1	theta	133	0.013	0.006	183	-0.009	0.005	-0.573	-0.121	-0.345	0.003	0.005
3	5.1	6.5	theta	133	0.010	0.007	183	-0.007	0.006	-0.458	-0.010	-0.232	0.041	0.048
4	6.5	8.3	theta	133	0.029	0.011	182	-0.021	0.009	-0.658	-0.201	-0.427	0.000	0.001
5	8.3	10.5	alpha	132	0.082	0.017	182	-0.058	0.014	-1.064	-0.581	-0.817	0.000	0.000
6	10.5	13.4	alpha	133	0.065	0.014	180	-0.047	0.011	-1.062	-0.578	-0.815	0.000	0.000
7	13.4	17.0	beta	133	0.033	0.007	183	-0.023	0.005	-1.069	-0.587	-0.823	0.000	0.000
8	17.0	21.7	beta	133	0.029	0.006	183	-0.021	0.005	-1.068	-0.586	-0.822	0.000	0.000
9	21.7	27.6	beta	133	0.011	0.005	180	-0.008	0.005	-0.577	-0.122	-0.347	0.002	0.005
10	27.6	35.2	gamma	133	0.003	0.007	180	-0.003	0.006	-0.307	0.141	-0.082	0.470	0.470
11	35.2	44.8	gamma	133	-0.007	0.009	177	0.005	0.008	-0.098	0.353	0.127	0.267	0.290
12	44.8	57.1	gamma	133	-0.020	0.010	177	0.015	0.009	0.095	0.551	0.321	0.005	0.009
13	57.1	72.7	gamma	133	-0.019	0.011	181	0.014	0.009	0.066	0.518	0.290	0.011	0.016

Supplementary Table 11. Covariate-adjusted group comparison statistics: Separation of high- and low-power oscillations ($E+I_{HLS}$).

Statistical analysis was identical to that described for Supplementary Table 7 (BiS). Reported statistics include group means (±SE) for HC and MDD, effect sizes as Cohen's *d* (MDD − HC), 95% confidence intervals for *d*, and *p*- and FDR-adjusted *p*-values.
